# Unraveling the Mechanism of StWRKY6 in Potato (*Solanum tuberosum*)’s Cadmium Tolerance for Ensuring Food Safety

**DOI:** 10.3390/foods12122303

**Published:** 2023-06-07

**Authors:** Guandi He, Muhammad Saleem, Tingfei Deng, Zhuoyan Zhong, Tengbing He, Jiahai Wu

**Affiliations:** 1 College of Agriculture, Guizhou University, Guiyang 550025, China; gdhe@gzu.edu.cn (G.H.); gs.zyzhong22@gzu.edu.cn (Z.Z.); tbhe@gzu.edu.cn (T.H.); 2 Guizhou Provincial Academy of Agricultural Sciences, Animal Husbandry and Veterinary Research Institute, Guiyang 550005, China; 3 Department of Biological Sciences, Alabama State University, Office 314, 1627 Harris Way, Montgomery, AL 36104, USA; msaleem@alasu.edu; 4 National Products Research Center of Guizhou Province, Guiyang 550025, China; dengtingfie@sina.com

**Keywords:** plant stress adaptation, WRKY transcription factor, reactive oxygen species scavenging enzyme, Cd-contaminated soil, crop genetic resources

## Abstract

The WRKY transcription factor plays a crucial role in plant stress adaptation. Our research has found that WRKY6 in *Solanum tuberosum* (potatoes) is closely related to cadmium (Cd) tolerance. Therefore, investigating the mechanism of StWRKY6 in plant resistance to Cd toxicity is of great scientific importance for food safety. This research further analyzed the gene structure and functional regions of the nuclear transcription factor WRKY6 in potatoes, discovering that StWRKY6 contains W box, GB/box, ABRE, and other elements that can act as a nuclear transcription regulatory factor to execute multiple functional regulations. The results of the heterologous expression of StWRKY6 in Arabidopsis under Cd stress showed that the overexpression line (StWRKY6-OE) had significantly higher SAPD values and content of reactive oxygen species scavenging enzymes than the wild type, indicating that StWRKY6 plays a crucial role in protecting the photosynthetic system and promoting carbohydrate synthesis. Transcriptome analysis also revealed that the Cd-induced expression of StWRKY6 up-regulated many potential gene targets, including APR2, DFRA, ABCG1, VSP2, ERF013, SAUR64/67, and BBX20, which are involved in Cd chelation (APR2, DFRA), plant defense (VSP2, PDF1.4), toxic substance efflux (ABCG1), light morphology development (BBX20), and auxin signal (SAUR64/67). These genes coordinate the regulation of Cd tolerance in the StWRKY6 overexpression line. In summary, this study identified a potential gene set of the co-expression module of StWRKY6, providing useful evidence for the remediation of Cd-contaminated soil and the genetic breeding of low Cd-accumulating crops, thereby ensuring food safety.

## 1. Introduction

WRKY proteins are a class of transcription factors (TFs) that widely exist in plants. [1]. They can recruit other transcriptional regulatory factors and RNA polymerases to form a transcriptional complex, thereby promoting or inhibiting the transcription of downstream genes [2,3]. Based on our previous research, we found that the expression of the potato (*Solanum tuberosum*) WRKY6 gene was closely related to plant tolerance to cadmium (Cd), suggesting its possible involvement in regulating the accumulation of Cd in plants [4,5]. Cd is a toxic heavy metal that widely exists in the natural environment. It can easily accumulate in the food chain, seriously affecting plant growth and food safety [6]. Therefore, an in-depth investigation of the mechanism of StWRKY6 in plant resistance to Cd toxicity is of great scientific significance for achieving sustainable agricultural production, ensuring food security, and reducing environmental pressure caused by Cd pollution.

The function and structural characteristics of StWRKY6 are also issues that we need to further explore. We will analyze its gene structure, identify potential functional regions and elements, and further elucidate its multiple regulatory mechanisms. WRKY TFs have two conserved domains, namely the N-terminal DNA binding domain and the C-terminal WRKY domain [7]. The gene structure of the potato nuclear transcription factor WRKY6 is similar to most WRKYs, containing these two domains [8]. In addition, our study has revealed that StWRKY6 contains cis-acting elements, such as W box, GB/box, and ABRE, which usually act as promoter elements in response to stress signals and play an important regulatory role in plant resistance to stress. Currently, it has been demonstrated that multiple TFs, such as AtMYB4 (*Arabidopsis thaliana* Myeloblastosis transcription factor 4) [9], ZIP8 (Zrt- and Irt-like protein 8) [10], StERF075/077/126 (*Solanum tuberosum* Ethylene Response Factor 075/077/126) [11], TtbHLH29 (*Triticum turgidum* L. subsp. *Durum* basic helix–loop–helix transcription factor 29) [12], and AtWRKY12 (*Arabidopsis thaliana* WRKY transcription factor 12) [13], participated in Cd detoxification. StWRKY6 may also serve as a nuclear transcriptional regulatory factor to perform various functional regulations and may involve the mutual intersection and regulation of multiple signaling networks. This further suggests the complex regulatory mechanism of StWRKY6 in plant resistance to Cd toxicity, emphasizing the importance of the in-depth study of its function and structural characteristics for understanding its stress adaptation mechanism and developing low Cd accumulation in Cd-tolerant crops.

The Cd toxicity regulatory network is a complex system, involving many genes that participate in plant responses and adaptations to Cd toxicity [14]. Among these genes, some have been proven to be associated with Cd resistance [15]. For example, APR2 (adenosine 5′-phosphosulfate reductase 2) and PCS (Phytochelatin Synthase) are responsible for chelating Cd [16,17]. They can bind Cd to form Cd–S chelates and enrich them in cell or extracellular tissues, thereby lowering the accumulation and toxicity of Cd ions. Other genes, such as PCR1 (plant cadmium resistance 1) and FC1 (Ferrochelatase 1) [18,19], are plant defense genes that can enhance plant resistance when subjected to stress. The proteins encoded by these genes can strengthen the plant’s antioxidant ability, improve its tolerance to adverse environments, and alleviate oxidative damage caused by Cd ions. Additionally, some genes such as ABCG1 (ATP-binding cassette sub-family G member 1) can promote the efflux of toxic substances, thus reducing Cd toxicity [20]. BBX20 (B-box family transcription factor 20) and SAUR56/60 (Small Auxin Up RNA 56/60) are involved in photomorphogenesis and auxin signaling, respectively, both of which are essential factors in plant growth and development [21,22]. Their regulation may also affect the response and adaptation of plants to Cd toxicity. The functions and interactions of these genes are extremely complex, and further research is needed to understand their regulatory networks and interactions, so as to enhance plant resistance to Cd toxicity and reduce its impact on the environment and human health.

In addition to the above-mentioned content, we also need to further explore the importance and advantages of heterologous expression experiments. Heterologous expression is the process of cloning a foreign gene into a host cell or organism and expressing it in that cell or organism [23,24]. Through heterologous expression, we can eliminate the influence of potato endogenous genes on the regulation of StWRKY6 expression, thus better studying the StWRKY6’s response to Cd stress and its impact on plant physiological and biochemical processes. At the same time, by conducting heterologous expression experiments in Arabidopsis, we can compare differences between different plants and investigate whether StWRKY6 has similar mechanisms of action, further understanding its functional and structural characteristics.

As a model plant, Arabidopsis has rich genomic annotation information and has been widely used in plant gene function and molecular mechanism research [23,25]. Hence, in transcriptome analysis and gene mining, Arabidopsis proves to be an ideal subject to identify the regulatory genes of StWRKY6 and their underlying mechanisms in plant resistance to Cd toxicity, thus ensuring food safety and security. By analyzing the genes regulated by StWRKY6 and their regulatory mechanisms in Arabidopsis, we can gain insights into the potential implications of StWRKY6 on food safety. Additionally, this research on StWRKY6’s role in plant resistance to Cd toxicity can be beneficial in finding novel strategies to mitigate Cd-contaminated soil, developing low Cd accumulation Cd-tolerant crops, and other important agricultural applications that promote food safety. Furthermore, through heterologous expression experiments and research using Arabidopsis as a model plant, we can extend our understanding of StWRKY6’s function and regulatory mechanisms, leading to effective measures for safe crop production and soil remediation.

If StWRKY6 is confirmed to be a positive regulator of plant tolerance to Cd and a negative regulator of Cd accumulation, enhancing StWRKY6 expression can increase plant resistance to Cd and reduce Cd accumulation in the edible parts of plants, which is crucial for ensuring food safety and security. We can use various physical or biochemical methods to enhance the transcription of the StWRKY6 gene and reduce Cd accumulation in the edible parts of crops, without resorting to genetic modification. Alternatively, in plants, we can develop regulators or germplasm resources that suppress the expression of WRKY6 homologs to create Cd-hyperaccumulating plants specifically for the Cd remediation of soil. Furthermore, we can explore a dual regulation pathway at the molecular level that can simultaneously increase Cd content in the roots, stems, and leaves (non-edible parts) of crops while reducing Cd accumulation in edible parts, thereby promoting food safety and utilizing non-edible plant parts for the recycling management of Cd in the soil. These strategies could be promising for safe crop production, and our research on StWRKY6’s role in plant resistance to Cd toxicity can also provide novel strategies to mitigate Cd-contaminated soil and other important agricultural applications.

In summary, to ensure food safety and security, we will use a variety of methods, including gene structure analysis, Arabidopsis heterologous expression analysis, transcriptome analysis, signal cascade analysis, etc., to further explore the mechanism of StWRKY6 in plant stress adaptation under Cd contamination. Through these methods, we can comprehensively understand the structural characteristics of StWRKY6 and further study its response to Cd stress and its impact on plant physiological and biochemical processes. Additionally, we will also use transcriptome analysis and gene mining technology to identify potential gene targets of StWRKY6, further investigate the regulatory effect of Cd on these genes, and unravel the mechanism by which these genes contribute to plant tolerance to Cd toxicity, taking into account their role in ensuring food safety and security. Accordingly, this research is expected to provide a theoretical basis for deepening our understanding of StWRKY6’s function and regulatory mechanisms and for developing low Cd accumulation in Cd-tolerant crops with vital agricultural application prospects, ultimately safeguarding food safety.

## 2. Results

### 2.1. Subcellular Localization of StWRKY6 in Tobacco Leaves

**Figure 1:**
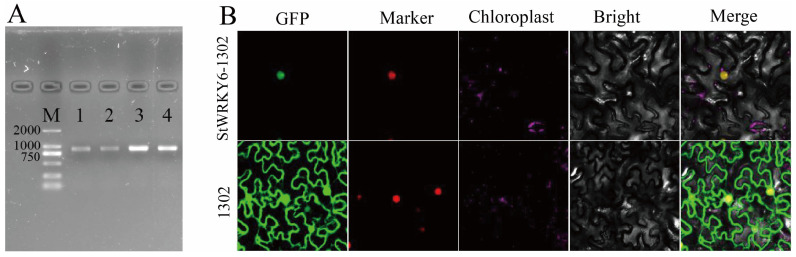
Cloning and subcellular localization of StWRKY6. (**A**) Gel electrophoresis results; M is Direct-Load Star Marker D2000; the brightest band of M is 750 bp; all of 1, 2, 3 and 4 represent StWRKY6. (**B**) Subcellular localization of StWRKY6, and the fluorescence channels of StWRKY6-1302 and control (empty vector 1302) from left to right are GFP, Marker, Chloroplast, Bright and Merge.

As shown in Figure 1A, the PCR amplification of StWRKY6 generated a band with a length of approximately 882 bp, which was close to the expected length of the target gene. The sequencing results of the StWRKY6 amplicon were compared to the reference CDS sequence, and the similarity was found to be over 90%, indicating that the PCR reaction was accurate and reliable.

As a widely used model plant species, tobacco (*Nicotiana benthamiana*) has been extensively studied and applied in many research fields. *Nicotiana benthamiana* (*N. benthamiana*) exhibits several advantageous features, including fast growth rate, easy transformation, and relatively simple genome sequencing, which makes it an ideal experimental plant model. Moreover, its small size and tender leaves make it easier for researchers to observe and conduct subcellular localization experiments. The choice of using *N. benthamiana* for our immunodetection analysis therefore allowed us to obtain high-quality data on the subcellular localization of StWRKY6 with greater accuracy. To further investigate the subcellular localization of StWRKY6, GV3101 strains containing StWRKY6-1302 plasmid or empty vector 1302 were mixed with GV3101 strains containing the marker at a 1:1 ratio and injected into *N. benthamiana* leaves. After two days of weak light incubation, the fluorescence signal was observed under a fluorescence microscope. The results showed that the fluorescence signal of StWRKY6-1302 fused protein was only detected in the nucleus of the tobacco leaves, while the control cells expressed with the empty vector displayed fluorescence signals throughout the whole cell (Figure 1B). These results indicate that StWRKY6 is located in the nucleus and functions as a nuclear transcription factor to regulate a series of complex transcription activation mechanisms.

### 2.2. StWRKY6 Protein Sequence and Cis-Acting Element Analysis

**Figure 2:**
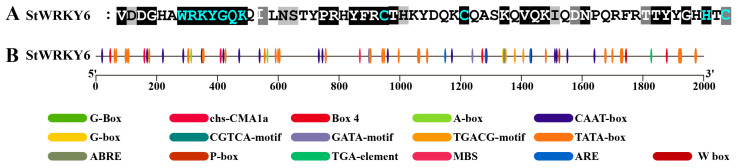
Sequence characterization of StWRKY6 protein and analysis of 2000 bp cis-acting elements upstream of the promoter. (**A**) Conserved sequence analysis of StWRKY6, the parts highlighted in blue are conserved WRKY domain and zinc finger structure. (**B**) Cis-acting elements analysis, each type cis-acting element is represented by an ellipse with a specific color, and the function of corresponding cis-acting element has been illustrated at the bottom of the picture.

As evidenced by Figure 2A, StWRKY6 is characterized by only having one WRKY domain—the “WRKYGQK” motif—and a zinc finger structure of C-X7-C-X23-H-X1-C (C2HC), thereby categorizing it as a III-class WRKY TF. Additionally, Figure 2B reveals that StWRKY6 contains multiple elements that are closely related to light response, such as G-box, chs-CMA1a, and box 4, as well as hormone response elements, including CGTCA-motif, ABRE, P-box, TGA-element, and transcription initiation regulation elements, such as TATA-box and CAAT-box. These findings indicate that StWRKY6 plays important roles in the growth and adaptation processes of potato plants, particularly in relation to environmental stress. This suggests that StWRKY6 may play a crucial role in aiding the potato plant’s ability to adapt to harsh environmental conditions.

### 2.3. Protein Interaction Analysis Revealed the Role of StWRKY6 in Potato Stress Adaptation

**Figure 3:**
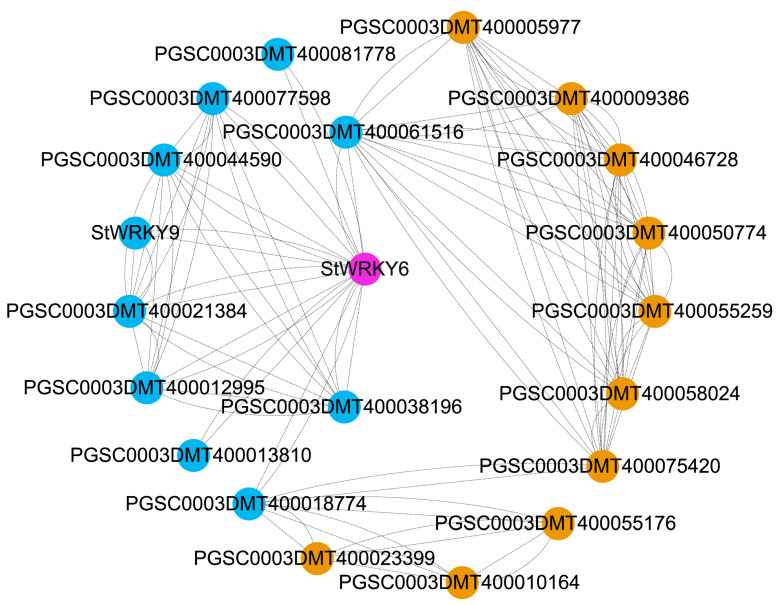
Interacting proteins of StWRKY6. The lines represent the interaction relationships between StWRKY6 and other proteins. The gene/protein names were extracted from the Potato Genome Sequencing Consortium database (http://spuddb.uga.edu/, accessed on 5 June 2023) PGSC DM v4.03 version, and relevant annotation information can be found on this database. The proteins directly interacting with StWRKY6 are marked in blue, and those indirectly interacting with it are marked in orange.

Stress-induced changes in gene expression are a fundamental mechanism that plants use to cope with various environmental stressors. In particular, TFs play a crucial role in modulating gene expression by either activating or inhibiting target genes in response to stress. Among these TFs, the members of the WRKY TF family are involved in regulating various stress-responsive genes in plants. StWRKY6 is a member of the WRKY family, and protein interaction analysis results show that StWRKY6 directly interacts with 10 proteins in the potato genome (Figure 3), most of which are known enzymes related to the plant stress response. For example, peroxidases are involved in detoxifying reactive oxygen species (ROS) produced during oxidative stress, while E3 ubiquitin ligases regulate protein degradation in response to stress. The direct interaction between StWRKY6 and these proteins further highlights the key role played by this TF in promoting potato growth under adverse environmental conditions. Additionally, the indirect protein interactions of StWRKY6 provide further insights into its potential biological functions in regulating various metabolic and physiological processes in potato. The proteins that may indirectly interact with StWRKY6 include those related to vacuolar proteins, acetylglutamate synthase, and chlorophyll fluorescence. These proteins are involved in various cellular processes such as protein transport, amino acid biosynthesis, and photosynthetic electron transport. By regulating the expression of genes associated with these processes, StWRKY6 might play a significant role in maintaining normal potato growth and development under different environmental conditions. It is worth noting that StWRKY6 also participates in regulating genes related to the PS II complex, which is critical for capturing light during photosynthesis. Changes in the stability of the PS II complex could significantly affect potato growth and development, particularly under stressful environments. Therefore, the critical role played by StWRKY6 in potato environmental adaptation and PS II stability highlights the importance of understanding its potential molecular mechanisms further. Protein interaction analysis provides new insights into the critical roles played by StWRKY6 in potato environmental adaptation and PS II stability. These findings also provide a reference for transcriptome and co-expression network analysis in the future.

### 2.4. Improving Photosynthesis and Reducing Cd Toxicity-Induced Chlorosis through StWRKY6 Overexpression

**Figure 4:**
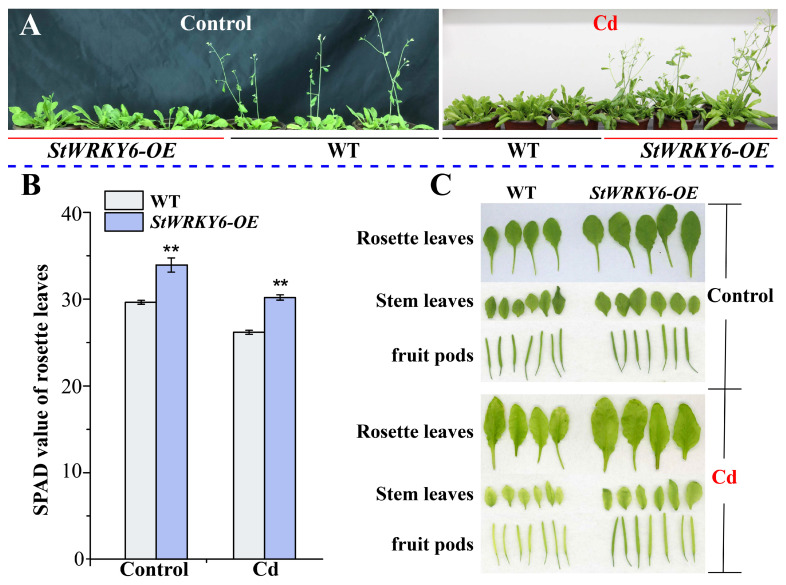
Phenotypic difference and changes in SPAD value between StWRKY6-0E and WT. (**A**) Difference in flowering time. (**B**) Changes in SPAD value (**: *p* < 0.01; Student’s *t*-test). (**C**) Difference in rosette leaves, stem leaves, and fruit pods. Control: 0 μmol/L CdCl_2_; Cd: 100 μmol/L CdCl_2_.

Research has shown that StWRKY6 is involved in the potato’s adaptation to environmental stress, regulating the expression of genes related to oxidative stress, protein degradation, and photosynthesis through direct and indirect interactions. It may also regulate gene expression related to other aspects of growth and development. Under control conditions, StWRKY6 overexpression lines had a later flowering period than wild-type plants, and the stems and pods were thicker than wild-type (Figure 4). However, to be more specific, both StWRKY6-OE and wild-type plants showed a visible change in leaf color under Cd toxicity treatment, indicating Cd-induced chlorosis symptoms. However, StWRKY6-OE plants showed less reduction in leaf greenness than wild-type plants. To quantify this observation, we analyzed the leaf color using the CIELAB method, which provides a more objective and accurate measurement of color changes. In addition, we measured SPAD values as a measure of chlorophyll content in flower ring leaves of both StWRKY6-OE and wild-type plants. Our results showed that the flower ring leaf SPAD content of StWRKY6-OE plants was significantly higher than that of wild-type plants, indicating that StWRKY6 plays a crucial role in protecting the photosynthetic system and promoting carbohydrate synthesis in transgenic plants. This result suggests that StWRKY6 may enhance plant stress tolerance by regulating gene expression, playing a key role in responding to various stresses. Furthermore, our study found that StWRKY6 overexpression can lead to increased flowering period, larger leaf size, and higher flower ring leaf SPAD content, which can improve plant tolerance and stress resistance. Thus, it has the potential to be valuable for agricultural production. However, there are still many questions that need to be studied further, such as StWRKY6’s precise regulatory mechanism, related signaling pathways, and interactions with other TFs. Answering these questions will help reveal the molecular mechanism of plant stress response, growth, and development, providing a theoretical basis and technical support for breeding high-yield, high-stress-resistant new crop varieties.

### 2.5. Enhanced Protective Enzyme Activity and Reduced MDA Content Contribute to StWRKY6-Mediated Cd Tolerance in Plants

SOD (superoxide dismutase), POD (peroxidase), and CAT (catalase) are important enzymes involved in the antioxidant defense system of plants. SOD converts superoxide radicals to hydrogen peroxide and oxygen, while POD and CAT remove hydrogen peroxide by converting it to water and oxygen. Their activities are closely linked to oxidative stress in plant cells, and the enzymes play a crucial role in reducing oxidative damage and increasing stress tolerance.

**Figure 5:**
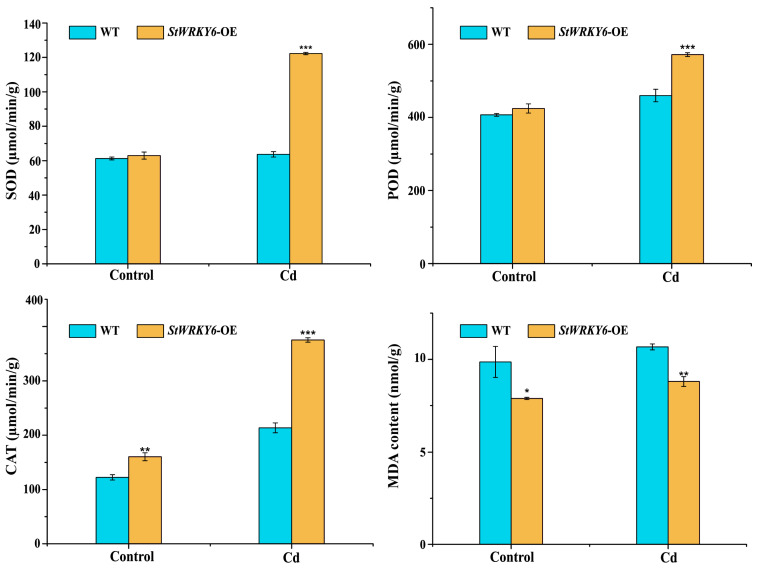
Detection of enzymatic activities and MDA content. The value of the ordinate is equal to the mean (±SD) of three biological replicates and is represented by the height of the bar graph. * *p* < 0.05, ** *p* < 0.01, *** *p* < 0.001. Control: 0 μmol/L CdCl_2_; Cd: 100 μmol/L CdCl_2_.

In Figure 5, our findings indicate that StWRKY6 overexpression led to a significant increase in CAT activity under control conditions (*p* < 0.01). This suggests that StWRKY6 may regulate the expression of catalase genes, which is crucial for maintaining redox homeostasis in plant cells. During Cadmium stress, the StWRKY6-OE plants showed a significant increase in SOD, POD, and CAT activity (*p* < 0.001) compared to the wild-type plants. This indicates that the overexpression of StWRKY6 can enhance the enzymatic activity of the antioxidant defense system, which leads to better ROS scavenging and reduced oxidative stress.

Furthermore, the significant decrease in MDA (malondialdehyde) content in StWRKY6-OE plants under Cd stress (*p* < 0.01) suggests that these plants experience less lipid peroxidation and membrane damage. Since MDA is a product of lipid peroxidation and a widely used indicator of oxidative damage, the lower levels of MDA in StWRKY6-OE plants indicate better protection against oxidative stress. This is consistent with the enhanced activity of SOD, POD, and CAT enzymes in these plants.

Taken together, these results suggest that StWRKY6 plays a critical role in regulating the antioxidant defense system and maintaining redox homeostasis in plants. The overexpression of StWRKY6 enhances the activity of protective enzymes, such as CAT, which leads to better ROS scavenging and reduced oxidative stress. The decrease in MDA content also indicates that the StWRKY6-OE plants are better protected against oxidative damage under Cd stress. In conclusion, our findings provide important insights into the role of StWRKY6 in plant stress adaptation and suggest that it could be a promising target for genetic engineering in improving plant stress tolerance and productivity. Therefore, the subsequent analysis of downstream target gene set based on StWRKY6 is expected to provide a potential target set for the interpretation of its function.

### 2.6. Transcriptional Regulation by StWRKY6 Reduces Cd Accumulation and Enhances Plant Tolerance

**Figure 6:**
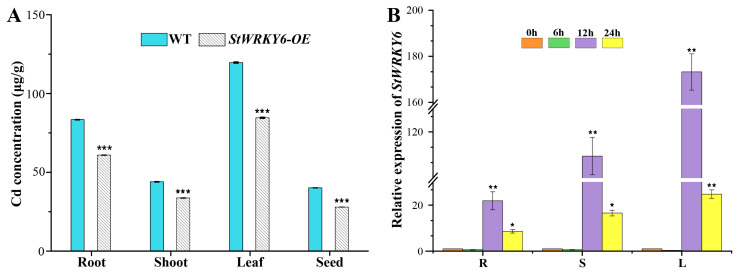
StWRKY6-OE mediates tissue-specific distribution of cadmium and spatiotemporal expression profiles in Arabidopsis.. (**A**) Determination of Cd in plant various tissues. The value is equal to the means (± SD) of three biological replicates and represented with the height of the bar graphs, which is the value of the expression. (**B**) Expression pattern of StWRKY6. The relative expression level is represented by the height of the bar graphs, where the value of the expression is equal to the means (±SD) of three biological replicates. * *p* < 0.05, ** *p* < 0.01, *** *p* < 0.001.

The results in Figure 6 demonstrate that StWRKY6 overexpression can enhance Cd tolerance in plants by reducing Cd accumulation and regulating gene expression under Cd stress. StWRKY6-OE plants showed significantly lower Cd concentrations in their roots, stems, leaves, and seeds compared to wild-type (WT) plants (*p* < 0.05), indicating that StWRKY6 overexpression can reduce Cd accumulation in plants. Moreover, qRT-PCR analysis results showed that StWRKY6 was highly expressed in the roots, stems, and leaves of StWRKY6-OE plants under Cd stress, with a similar temporal pattern of expression (6 h < 12 h < 24 h). The highest expression levels of StWRKY6 were observed at 12 h under Cd stress, which were 21.89-fold, 109.52-fold, and 173.23-fold higher than at 0 h in the roots, stems, and leaves, respectively. These results suggest that StWRKY6 plays a crucial role in responding to Cd stress by regulating gene expression related to Cd detoxification.

Taken together, these findings suggest that StWRKY6 overexpression can reduce Cd accumulation in plants and enhance Cd tolerance by regulating gene expression related to Cd detoxification. This highlights the potential of StWRKY6 as a target for genetic engineering to improve plant Cd tolerance and productivity in contaminated soils. However, further research is needed to fully elucidate the regulatory mechanisms of StWRKY6 and its interactions with other factors involved in Cd transport and detoxification in plants.

### 2.7. Elucidating the StWRKY6-Mediated Cd Detoxification Pathway in Transgenic Arabidopsis through RNA-seq and GO Enrichment Analysis

**Figure 7:**
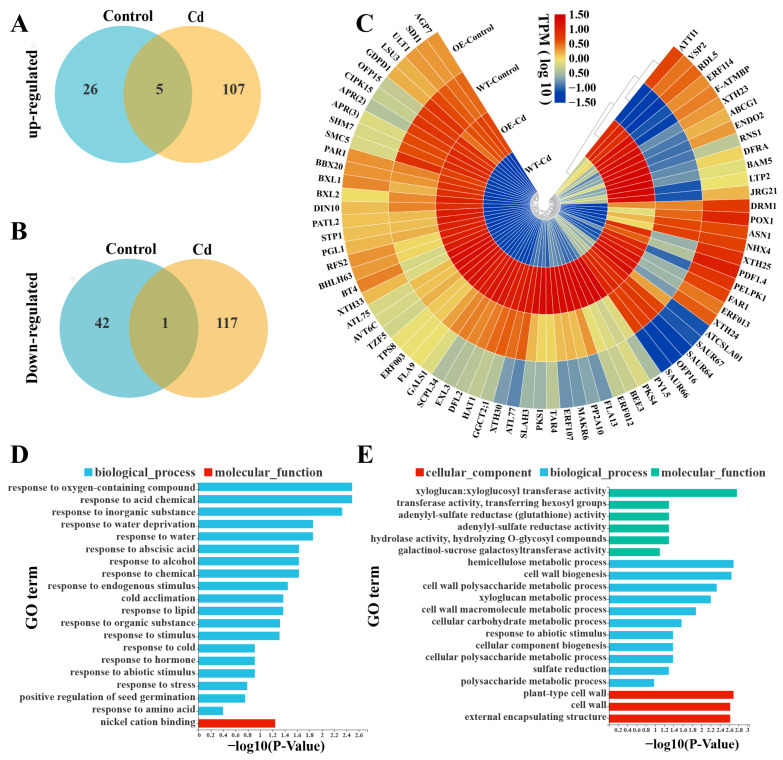
Differential gene expression profile of StWRKY6-OE leaves in control and Cd stress compared with WT revealed a set of genes that collectively responded to Cd poisoning. (**A**,**B**) Venn diagrams showing the numbers of genes up-regulated and down-regulated by StWRKY6-OE in both control and Cd treatment compared with WT. (**C**) Heat map of differentially up-regulated genes in StWRKY6 under Cd stress compared with WT. (**D**,**E**) The top 20 gene ontology (GO) terms for the commonly up-regulated genes of StWRKY6-OE in the control and Cd stress compared with WT.

To investigate the downstream regulatory gene set potentially controlled by StWRKY6 in Cd detoxification, we performed RNA-seq analysis on StWRKY6-OE and WT plants grown in cadmium-contaminated soil. Our RNA-seq analysis revealed 74 differentially expressed genes (DEGs) in control conditions, of which 31 were up-regulated and 43 were down-regulated. However, under Cd treatment, a total of 230 DEGs were observed, with 112 genes showing up-regulation and 118 genes showing down-regulation (*p* < 0.05). These results are presented in Figure 7A,B.

The metabolic pathway analysis of the DEGs revealed changes in several pathways related to Cd detoxification and stress response as a result of StWRKY6 overexpression. Moreover, the interaction between differentially up-regulated genes was investigated, revealing a complex network of regulatory interactions among these genes (Figures S2 and S3).

We conducted gene ontology (GO) enrichment analysis (Figure 7D,E) on the DEGs to gain insight into the mechanisms underlying StWRKY6-mediated growth and morphogenesis in transgenic plants and tolerance to Cd stress. Under control conditions, the up-regulated genes were mainly associated with oxygen-containing compounds, acid chemicals, stimuli, and abiotic stimuli. In contrast, under Cd stress, the up-regulated genes were primarily involved in xyloglucosyl transferase activity, hemicellulose metabolic processes, and cell wall polysaccharide metabolic processes. Notably, several up-regulated genes were closely related to oxygenated compounds, stress, cell wall polysaccharide metabolic processes, and hemicellulose metabolism.

We generated the expression heat maps of the differentially up-regulated genes in StWRKY6-OE plants compared to WT under Cd stress treatment (Figure 7C). The analysis revealed the differential expression of transcription factors (such as ERFs, XTHs, and BHLH63), auxin-induced SAURs, and adenosine 5′-phosphosulfate reductase 2 (AP2). Among these, several genes, including XTH24 to XTH25 and JGR21 to ATTI1, were significantly up-regulated in StWRKY6-OE plants compared to WT under both control and Cd treatment conditions. Conversely, SAUR64 to ATCSLA01 were significantly down-regulated in StWRKY6-OE plants compared to WT under Cd stress.

To further elucidate the mechanisms underlying the StWRKY6-mediated Cd detoxification pathway in transgenic Arabidopsis, we identified a set of co-expressed genes and a set of negatively regulated genes. These gene sets are presented in Table S3A–F. Our findings suggest that StWRKY6 overexpression can actively regulate growth and development in transgenic plants while enhancing their tolerance to Cd stress by modulating the expression levels of relevant genes. In summary, our study identified co-expressed and negatively regulated gene sets through RNA-seq and GO enrichment analysis, shedding light on the StWRKY6-mediated Cd detoxification pathway in transgenic Arabidopsis. Our results showed that StWRKY6 overexpression can enhance Cd tolerance in plants by affecting the expression of relevant genes. These gene sets have the potential to become novel targets for genetic engineering aimed at increasing plant resistance to heavy metal stress, with significant implications for sustainable agriculture.

## 3. Discussion

### 3.1. StWRKY6 Regulates Cd Tolerance in Arabidopsis

Cd is an inevitable environmental pollutant that severely affects plant growth and development [26]. In this study, we found that StWRKY6 is an important transcription factor that can increase the Cd tolerance of Arabidopsis. Furthermore, we also found that the overexpression of StWRKY6 significantly reduces the Cd content in different parts of transgenic Arabidopsis. This suggests that StWRKY6 plays a key role in regulating the plant’s response and process to Cd.

Previous studies have shown that some protein families play important roles in plant adaptation to heavy metal pollution. For example, ZAT10 protein in Arabidopsis increased cadmium tolerance by inhibiting IRT1 gene transcription and regulating the expression of four heavy metal detoxification genes [27]. In comparison, our study found that StWRKY6 can also significantly increase the Cd tolerance of Arabidopsis, but its regulatory mechanism is slightly different from ZAT6. Specifically, StWRKY6 can directly bind to the promoter regions of multiple TFs and downstream response genes, thereby affecting their expression levels. These downstream response genes not only include metabolism-related genes, oxidoreductases, and ion transport-related genes but also involve protein degradation and other aspects. The regulation of these genes promoted the production of more antioxidants, enhanced photosynthesis, and carbon metabolism, thereby increasing the Cd tolerance of Arabidopsis, which has crucial implications for food safety. Our research findings further demonstrated that the overexpression of StWRKY6 reduced the accumulation of Cd in plants, highlighting the critical role of StWRKY6 in regulating Cd tolerance in Arabidopsis. These results have promising applications for developing agricultural strategies that ensure the safe production of crops and promote food security.

### 3.2. StWRKY6 Reduces Cadmium-Induced Oxidative Stress by Enhancing Antioxidant Enzyme Activity

Oxidative stress response is a common plant adaptive mechanism that is easily triggered under environmental stresses such as Cd [28]. This study shows that the overexpression of StWRKY6 can significantly increase the activity of protective enzymes (SOD, POD, and CAT) in Arabidopsis, while reducing the MDA content in plants. These results suggest that StWRKY6 can reduce cadmium-induced oxidative stress by enhancing the activity of antioxidant enzymes, thus improving plant tolerance.

Previous studies by Mahsa Modareszadeh et al. have shown that the overexpression of CAX3 may enhance Cd tolerance in plants by decreasing Cd-induced ROS production through activating antioxidant enzymes and intervening in the positive feedback loop between ROS generation and Cd-induced cytoplasmic Ca^2+^ spikes [29]. Similarly, our study also found that StWRKY6 can reduce cadmium-induced oxidative stress by enhancing the activity of protective enzymes but with the following differences: firstly, we further analyzed the regulatory mechanism of StWRKY6 on antioxidant enzyme genes. We found that the overexpression of StWRKY6 can significantly increase the activity of SOD, POD, and CAT in Arabidopsis, and the expression levels of these genes are closely related to the transcriptional regulatory activity of StWRKY6. This result provides a new way to explore the molecular mechanism of oxidative-reduction reaction in plants under heavy metal pollution. Secondly, we also found that the regulatory effect of StWRKY6 on Arabidopsis photosynthesis can help to reduce cadmium-induced oxidative stress. The promotion of photosynthesis can increase the ATP and NADPH content in plants, thus enhancing energy metabolism stability and further enhancing the activity of protective enzymes to reduce oxidative stress damage. These findings provide valuable references for further studying the molecular mechanism of StWRKY6, regulating the oxidation–reduction reaction in Arabidopsis.

### 3.3. Alleviating Cd Toxicity in StWRKY6 Transgenic Arabidopsis by Regulating Photosynthesis

Photosynthesis is an important process for plant growth and development, but heavy metal pollution such as Cd often inhibits plant photosynthesis [30]. In this study, we found that the overexpression of StWRKY6 can increase the SPAD value and flowering stage of Arabidopsis, which suggests that StWRKY6 may alleviate the toxicity of Cd in transgenic Arabidopsis by regulating photosynthesis. Some plant genes have been found to improve plant tolerance to heavy metals by regulating photosynthesis. MicroRNA (miR156) enhances plant Cd tolerance by regulating Cd uptake, transport, and detoxification, as well as reducing oxidative stress and damage to photosynthesis [31]. However, in comparison with previous studies, our research found that StWRKY6 can relieve cadmium-induced toxicity through the following two new pathways.

Firstly, our research findings showed that the overexpression of StWRKY6 increased the SPAD value and flowering stage of Arabidopsis, implying that StWRKY6 may promote photosynthesis by increasing the content of photosynthetic pigments or optimizing their distribution in leaves. This result highlights the critical relationship between the regulatory mechanism of StWRKY6 and heavy metal tolerance, providing novel strategies for overcoming Cd pollution problems and ensuring food safety. Secondly, we also discovered that the overexpression of StWRKY6 elevated the NADPH and ATP content in Arabidopsis leaves, which play a vital role in energy transfer and production, thereby promoting photosynthesis. Although NADPH is a substrate for the oxidation–reduction reactions, it also plays an essential role in Arabidopsis photosynthesis. This result suggests that StWRKY6 not only regulates metabolism-related genes but also regulates photosynthesis-related genes, thus enhancing plant tolerance to Cd and ultimately promoting food safety.

### 3.4. StWRKY6 as a Gene Regulatory Target to Enhance Plant Heavy Metal Tolerance

Gene regulation is a promising approach for improving plant tolerance and ensuring food safety. Our research findings have shown that StWRKY6 enhanced the tolerance of transgenic Arabidopsis to cadmium, highlighting its potential as a gene regulatory target for mitigating heavy metal pollution in crops. Future research can focus on studying the interaction between StWRKY6 and other genes, as well as exploring the response of StWRKY6 under various stress conditions. Such studies can provide a deeper understanding of the regulatory mechanism of StWRKY6 in plant stress adaptation and contribute to the development of innovative strategies for enhancing agricultural productivity while ensuring food safety and security.

Previous studies have shown that a TF MYB49 positively regulates the expression of Cd uptake-related genes and Cd accumulation, while ABA-induced ABI5 interacts with MYB49 to inhibit its downstream gene regulation, thereby reducing Cd accumulation [32]. However, our research found that StWRKY6 directly binds to the promoter region of downstream responsive genes, changing their expression levels, and increasing the tolerance of plants to cadmium. This discovery is different from previous studies and highlights the effectiveness of StWRKY6 as a gene regulatory target. Through transcriptome and gene expression profile analysis tools, we also identified the enrichment patterns and regulatory networks of these downstream responsive genes, further demonstrating the potential of StWRKY6 in improving plant tolerance to heavy metals. Moreover, we found that StWRKY6 is highly conserved in various plants, including important crops such as rice, wheat, and corn, indicating that StWRKY6 could be a universal and sustainable approach for enhancing the tolerance of different species and varieties to heavy metals. This research provides valuable insights into the response mechanism of plants to environmental stress and exploring plant biological resources, which has crucial implications for ensuring food safety and security.

## 4. Conclusions

**Figure 8:**
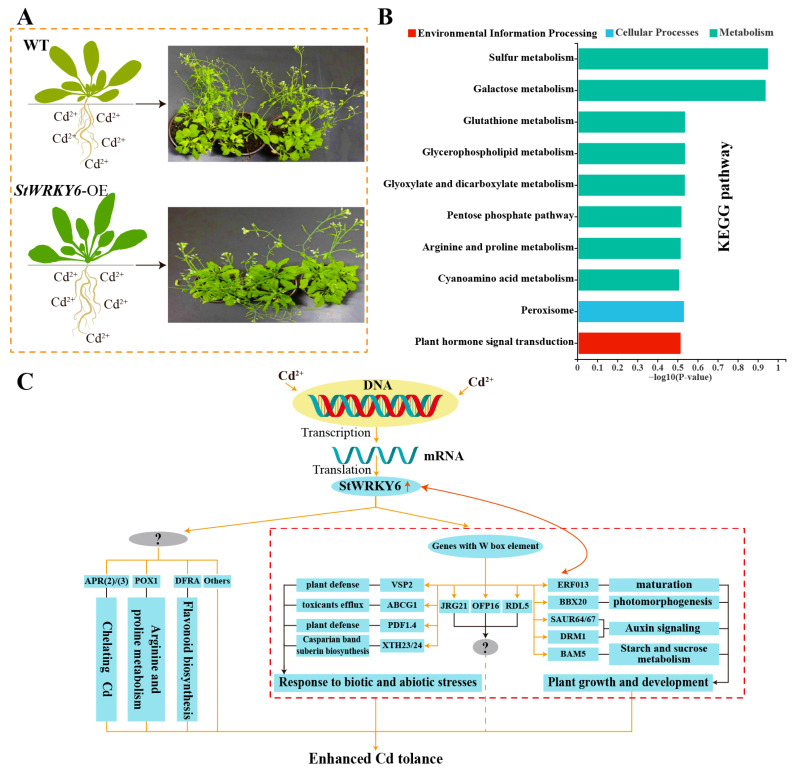
StWRKY6 enhanced the Cd tolerance. (**A**) Phenotypic difference of WT and StWRKY6-OE in 100 μmol/L CdCl_2_ treatment condition. (**B**) The top 10 KEGG pathways of differentially up-regulated genes in StWRKY6-OE under Cd treatment condition. (**C**) Model of the potential regulation mechanisms of StWRKY6 involved in Cd detoxification.

Our research findings have demonstrated that StWRKY6, a member of the WRKY Group III, plays a vital role in plant response to environmental stress. Through its interaction with different cis-acting elements, StWRKY6 regulates the expression of multiple genes, enhancing plant tolerance to toxic metals such as cadmium. This mechanism promotes antioxidant production, enhances photosynthesis and carbon metabolism, and increases NADPH and ATP content in Arabidopsis leaves. Furthermore, our study found that the overexpression of StWRKY6 can reduce the accumulation of cadmium in edible plant parts, highlighting its potential as a gene regulatory target for developing low cadmium accumulation crops with important agricultural applications. The conservation of StWRKY6 across multiple plant species, including important crops such as rice, wheat, and corn, makes it a promising approach for improving the tolerance of various species and varieties to heavy metals. Our findings provide valuable insights into the molecular mechanisms of plant response to environmental stress and offer effective strategies for mitigating heavy metal pollution and ensuring food safety and security. Please refer to Figure 8 for the specific potential regulatory model.

## 5. Materials and Methods

### 5.1. Plant Material, Treatment, and Analysis of Cd Accumulation and Enzyme Activity

The availability of plant materials: Seeds of Columbia wild-type Col-0 (WT) and StWRKY6-overexpressing (StWRKY6-OE) *Arabidopsis thaliana* plants in the T3 generation were obtained from the New Rural Development Institute of Guizhou University, Guizhou Province (26°27′13″ N, 106°39′22″ E), where all experiments were conducted. The StWRKY6-OE line was generated by introducing the StWRKY6 gene into Arabidopsis WT plants using the Agrobacterium-mediated floral dip method, followed by selection on 1/2 MS medium containing 50 μg/mL kanamycin. The T3 seeds were collected from self-pollinated homozygous plants and further confirmed by PCR using specific primers targeting the StWRKY6 gene.

Plant materials and growth conditions: Columbia wild-type (WT) and StWRKY6-overexpressing (StWRKY6-OE) Arabidopsis seeds were used for sowing. After sowing, the seedlings were grown in an RG × 400E artificial climate box with a light cycle of 16:00 to 8:00 the next day, at a temperature of 22.0 °C and a humidity of 50% rH, with a light intensity of 6 lux. When the plants grew four leaves, three plants were transplanted into flowerpots with diameters of 88, 80, and 60 mm and a height of 80 mm.

Experimental treatment and sample collection: WT and StWRKY6-OE plants were subjected to Cd stress by adding 60 mL of 100 μmol/L CdCl_2_ solution separately into their growth substrates (not adhering to the leaves). Distilled water was added as a control. After 12 h of Cd treatment, fresh leaf samples were collected and stored at −80 °C. A total of 12 samples were collected, and three biological replicates were performed for each treatment. The collected samples were transported on dry ice to Majorbio (Shanghai) for RNA-seq analysis.

Cd content measurement: One week after Cd treatment, samples of different tissue types (roots, stems, leaves, and seeds) from WT and StWRKY6-OE plants were collected to measure their Cd content. To determine the Cd content in various tissues of Arabidopsis, we used an HNO_3_-Automatic Graphite Digestion Apparatus-Inductively Coupled Plasma Optical Emission Spectrometer (ICP-OES-7000, Thermo Fisher Scientific, New York, USA), following the method described by Lou et al. [33]. As a quality control measure, we used a standard material (GBW10045a) alongside the plant samples.

The measurement of antioxidant enzyme activity and MDA content: Fresh leaf samples were collected one week after Cd treatment for the measurement of superoxide dismutase (SOD), peroxidase (POD), catalase (CAT), and malondialdehyde (MDA) levels using the BC0175-100, BC0095-100, BC0205-100, and BC0025-100 assay kits (Solarbio), respectively. Specifically, we followed the detection methods provided by the manufacturer for each kit.

### 5.2. Cloning and Subcellular Localization of StWRKY6

To investigate the subcellular localization of StWRKY6, we amplified the coding sequence of StWRKY6 without the stop codon from potato cDNA preserved at −80 °C, using the primers listed in Table 1. The EZ-Flex One Step Seamless Cloning Kit (GeneStar, Beijing Kangruncheng Biotechnology Co., Ltd., Beijing, China) was used to clone StWRKY6 into pCAMBIA1302 (1302), a plant dual expression vector containing the enhanced green fluorescent protein (mfGFP5) gene under the control of the CaMV 35S promoter. The construction of the StWRKY6-1302 vector is illustrated in Figure S1. The empty vector 1302-GFP was used as the control. The recombinant plasmids were transiently expressed in *Nicotiana benthamiana* leaves by Agrobacterium (GV3101) mediation. Fluorescent signals were detected using a Nikon C2 (Nikon, Japan) confocal microscopy imaging system, with mKate and NLS used as fusion fluorescent protein and nuclear localization protein, respectively.

### 5.3. Sequence and Cis-Acting Element Analysis of StWRKY6

The sequence analysis was conducted using GeneDoc (v3.2) to perform the multiple sequence alignment and visualization of DNA or protein sequences. To identify cis-acting elements present in the promoter region of StWRKY6, we utilized PlantCARE [34], an online software tool that specifically analyzes cis-acting regulatory plant elements. We obtained the 2000 bp sequence located upstream of the ATG start codon by retrieving it from NCBI [35] using the gene ID (StWRKY6: LOC102577893). Finally, for graphical visualization and analysis, we used TBtools (v1.0692) [36], a software package providing a range of tools for the analysis and visualization of genomics data. Identify potential regulatory elements involved in the transcriptional regulation of StWRKY6.

### 5.4. Interaction Proteins Analysis of StWRKY6

When using STRING (https://cn.string-db.org, accessed on 5 June 2023) [37] to analyze the interaction proteins of StWRKY6, we set *Solanum tuberosum* as the organism of interest and used a medium confidence score threshold of 0.4 to filter potential interacting partner proteins. This score threshold provides a balance between specificity and sensitivity in retrieving relevant protein–protein interactions. For the visualization analysis by Cytoscape (v3.6.1) [38], we imported the data from STRING and used various layout algorithms to generate different network visualizations that highlight the relationships between StWRKY6 and its interacting partners. Through careful selection and analysis of these interactions, we were able to gain new insights into the potential functions and regulatory mechanisms of StWRKY6 in potato plants.

### 5.5. Plant Phenotypic Analysis between Treatments

To induce Cd stress, we poured 60 mL of a CdCl_2_ solution (100 μmol/L) into flowerpots that were planted with WT and StWRKY6-OE plants. As a control, we added an equal volume of distilled water to separate pots. Throughout the experiment, we monitored the phenotype of the plants at each stage using both a camera and an A4-format image scanner (Epson Perfection V850 Pro, Shanghai, China). These images were then analyzed using WinRHIZO Pro 2019 image analysis software to quantify root morphology, including root length, surface area, and diameter. By comparing the results from the Cd-stressed plants against the control plants, we were able to determine how Cd stress affected the growth and development of the WT and StWRKY6-OE plant lines.

### 5.6. RNA Extraction and RT-qPCR Analysis

To study the temporal expression pattern of StWRKY6 in response to Cd stress, we subjected the plants to 60 mL of CdCl_2_ (100 μmol/L) stress for 0 h, 6 h, 12 h, and 24 h. At each time point, we harvested roots, stems, and leaves from the plants, then rinsed them with tap water three times and distilled water two times before absorbing surface moisture with absorbent paper. All samples were stored at −80 °C in an ultra-low temperature refrigerator until further analysis.

cDNA stands for “complementary DNA”, which is synthesized from RNA using reverse transcriptase enzyme in a process known as cDNA synthesis. This synthesized cDNA serves as a template for the amplification of specific genes of interest via PCR or reverse transcription-quantitative PCR (RT-qPCR) analysis. In our study, we used StarScript II First-strand cDNA Synthesis Mix, and 2 × RealStar Green Fast Mixture purchased from Kangruncheng Biotechnology Co., Ltd. (Beijing, China) to synthesize cDNA for the RT-qPCR analysis of gene expression. The RT-qPCR reaction volume was 20 μL, consisting of 7 μL of ultrapure water (Type I or Type II according to ISO or Grade I or II according to ASTM), 2 μL of cDNA, 1 μL of primers (0.5 μL each of forward and reverse primers), and 10 μL of 2 × RealStar Green Fast Mixture. The qRT-PCR was conducted using a BIO-RAD CFX96 (Bio-Rad, Hercules, CA, USA) and a two-step method experiment, with parameters set to 95 °C for 3 min, 95 °C for 15 s, and 60 °C for 20 s, repeated for 40 cycles. The relative expression level of StWRKY6 was calculated using the 2^−ΔΔCt^ method [39]. The primer sequences used for qRT-PCR amplification are shown in Table 1.

### 5.7. Analysis of RNA Sequencing Data

We utilized the TAIR10 *Arabidopsis thaliana* genome (http://plants.ensembl.org/Arabidopsis_thaliana/Info/Index, accessed on 5 June 2023) as a reference for transcriptomic analysis [40]. All clean reads were then aligned with this reference using Hisat2 (http://ccb.jhu.edu/software/hisat2/index.shtml, accessed on 5 June 2023) [41]. RSEM (http://deweylab.github.io/RSEM/, accessed on 5 June 2023) [42] was employed for the downstream quantification of gene and transcript expression levels, measured in TPM (transcripts per million). To identify differentially expressed genes between samples, we used DESeq2 [43]. The default parameters were set at *p*-adjust < 0.05 and |log2FC| ≥ 1 to ensure statistical significance and a meaningful difference in gene expression levels.

### 5.8. Subsection

To evaluate the impact of two stress levels and two cultivars on the morphological and physiological parameters, we employed a one-way analysis of variance (ANOVA). Multiple comparisons were performed using the LSD and Duncan’s tests. The values plotted on the bar graph represent the averages of three biological replicates (±SD), and statistical significance is indicated by * *p* < 0.05, ** *p* < 0.01, or *** *p* < 0.001.

We used Origin 9.0 and Cytoscape (v3.6.1) for drawing and Adobe Illustrator CC 2019 for graphic editing. This approach allowed us to generate visually appealing and informative graphs, providing a clear representation of the effects of various treatments on morphological and physiological parameters in the studied cultivars.

## Figures and Tables

**Table 1:** Primers of StWRKT6 for RT-qPCR and cloning.

Primer Name	Reverse Primer (5′–3′)	Forward Primer (5′–3′)
StWRKY6 (qRT-PCR)	CTTGACCTCAACCGGCGTAGTG	AACCTCTTGTTGGTGGTGGTGAAG
StWRKY6 (1302)	agttcttctcctttactagtCACTTGATCAAAATTCCAAAGACC	acgggggactcttgaccatggATGGATAACTCATCGACTG

## Data Availability

The original contributions presented in the study are included in the article/Supplementary Materials. Further inquiries can be directed to the corresponding authors.

## Supplementary Materials

The following supporting information can be downloaded at: https://www.mdpi.com/article/10.3390/foods12122303/s1, Table S1: Annotation of StWRKY6 interacting proteins and their information in the potato genome; Table S2: Analysis of cis-acting elements of up-regulated genes induced by StWRKY6-OE; Table S3: DEGs information of StWRKY6-OE and WT (see Table S3A–F for different groups); Table S3A: Genes differentially up-regulated in response to Cd treatment in StWRKY6-OE line; Table S3B: Differentially up-regulated genes in StWRKY6-OE line under non-Cd stress conditions; Table S3C: Genes differentially up-regulated in StWRKY6-OE line under both non-Cd and Cd stress conditions; Table S3D: Genes differentially down-regulated in StWRKY6-OE line under Cd stress conditions; Table S3E: Genes differentially down-regulated in StWRKY6-OE line under non-Cd stress conditions; Table S3F: Genes differentially down-regulated in StWRKY6-OE line under both non-Cd and Cd stress conditions; Figure S1: Details of StWRKY6-1302 vector; Figure S2: The ipath metabolic pathway analysis of differentially up-regulated genes in StWRKY6-OE compared with WT under Cd treatment; Figure S3: The interaction between differentially up-regulated genes in StWRKY6-OE under 100 μmol/L Cd treatment.
